# CRISPR/Cas9 genome-wide loss-of-function screening identifies druggable cellular factors involved in sunitinib resistance in renal cell carcinoma

**DOI:** 10.1038/s41416-020-01087-x

**Published:** 2020-09-24

**Authors:** Peter Makhov, Ji A. Sohn, Ilya G. Serebriiskii, Rushaniya Fazliyeva, Vladimir Khazak, Yanis Boumber, Robert G. Uzzo, Vladimir M. Kolenko

**Affiliations:** 1 Molecular Therapeutics Program, Fox Chase Cancer Center, Philadelphia, PA USA; 2 Blood Cell Development and Function Program, Fox Chase Cancer Center, Philadelphia, PA USA; 3 Institute of Fundamental Medicine and Biology, Kazan (Volga Region) Federal University, Kazan, Russian Federation; 4 Cancer Biology Program, Fox Chase Cancer Center, Philadelphia, PA USA; 5 Priaxon Inc., Philadelphia, PA USA; 6 Department of Hematology/Oncology, Fox Chase Cancer Center, Philadelphia, PA USA; 7 Division of Urologic Oncology, Department of Surgery, Fox Chase Cancer Center, Philadelphia, PA USA

**Keywords:** Urological cancer, Renal cell carcinoma

## Abstract

**Background:**

Multi-targeted tyrosine kinase inhibitors (TKIs) are the standard of care for patients with advanced clear cell renal cell carcinoma (ccRCC). However, a significant number of ccRCC patients are primarily refractory to targeted therapeutics, showing neither disease stabilisation nor clinical benefits.

**Methods:**

We used CRISPR/Cas9-based high-throughput loss of function (LOF) screening to identify cellular factors involved in the resistance to sunitinib. Next, we validated druggable molecular factors that are synthetically lethal with sunitinib treatment using cell and animal models of ccRCC.

**Results:**

Our screening identified farnesyltransferase among the top hits contributing to sunitinib resistance in ccRCC. Combined treatment with farnesyltransferase inhibitor lonafarnib potently augmented the anti-tumour efficacy of sunitinib both in vitro and in vivo.

**Conclusion:**

CRISPR/Cas9 LOF screening presents a promising approach to identify and target cellular factors involved in the resistance to anti-cancer therapeutics.

## Background

Renal cell carcinoma (RCC) is the most common type of kidney cancer with rising incidence.^1^ It is categorised into various subtypes, with clear cell RCC (ccRCC) representing ~85% of all RCC tumours.^2^ Papillary RCC and chromophobe RCC represent the most common remaining histologic subtypes with an incidence of 7–14% and 6–11%, respectively.^2^ Current targeted molecular strategies, including multitargeted tyrosine kinase inhibitors (TKIs), have resulted in a doubling of progression-free survival and significant gains in overall survival, thereby notably changing the treatment paradigm of advanced kidney cancer.^3,4^ Yet, about one-quarter of the ccRCC patients are primarily refractory to treatment with TKIs.^5^ Furthermore, most patients that respond initially will typically progress within 12 months of starting therapy.^6^

The ability of sunitinib to inhibit angiogenesis is well-established. However, we and others have demonstrated that, at concentrations found in human tumour specimens,^7^ sunitinib may also manifest a direct suppressive effect on tumour cells of various origins.^8–11^ Studies by Hillman et al. indicate that sunitinib exerts a direct cytotoxic effect on RCC cells at doses >0.5 μM.^12^ Concentrations of sunitinib in human tumour specimens can reach 9.5 + 2.4 μmol/L, whereas plasma concentrations were found to be significantly lower, 0.3 + 0.1 μmol/L.^11^ These findings suggest that sunitinib may selectively accumulate in tumour tissue at high concentrations and exert direct cytotoxic effect on tumour cells. Furthermore, some clinical studies suggest that the high response rate may result from a direct effect of TKIs on malignant cells.^13^

Protein farnesylation, catalysed by protein farnesyltransferase (FTase), plays important roles in the membrane association and protein–protein interaction of a number of eukaryotic proteins.^14^ FTase is located in the cell cytosol, and it is one of the three enzymes in the prenyltransferase group that catalyses most isoprenylation reactions. FTase adds a 15-carbon isoprenoid lipid (the farnesyl group) to proteins bearing a CAAX motif.^15^ Preclinical studies in the 1990s demonstrated that FTase inhibitors (FTIs) could successfully kill cancer cells both in vitro and in vivo with very little toxicity, thus generating much excitement toward the development of FTIs-based anti-cancer therapeutic regimens.^16^ Unfortunately, in most clinical trials, FTIs as monotherapy have not been as successful as expected.^16–19^ However, a combination of FTIs with cytotoxic agents improved the responses of patients with locally advanced breast cancer and some other advanced solid tumours.^16,20–22^

Synthetic lethality screens hold great promise for the development of novel therapeutic interventions. We have applied CRISPR/Cas9-based high-throughput loss-of-function (LOF) screening to identify genes involved in the resistance to sunitinib, a standard front-line therapeutic agent for the treatment of advanced ccRCC. Our search identified FTase and its downstream effectors among the top hits. Treatment of the sunitinib-resistant 786-O and PNX0010 ccRCC cells^9,23^ with FTI lonafarnib potently augmented the in vitro anti-tumour efficacy of sunitinib. Moreover, combined treatment with lonafarnib circumvented resistance to sunitinib in the PNX0010 xenograft tumour model. Therefore, concomitant treatment with lonafarnib and sunitinib may represent a rational therapeutic strategy for ccRCC patients with sunitinib-resistant tumours.

## Methods

### Cells and culture conditions

The 786-O human RCC cell line was obtained from ATCC. PNX0010 ccRCC cell line, which was described previously,^10,24^ was established from a fresh tumour specimen obtained intraoperatively from an RCC patient, undergoing nephron-sparing surgery at Fox Chase Cancer Center. This cell population is clinically correlated to an aggressive variant of ccRCC. PNX0010 cells are VHL-negative and express SETD2, BAP1 and PBRM1 proteins (Supplementary Fig. S1). Initial stocks were cryopreserved, and at every 6-month interval a fresh aliquot of frozen cells was used for the experiments. No authentication was done by the authors. Cells were cultured in RPMI 1640 (Bio-Whittaker) supplemented with 10% FCS (Hyclone), gentamicin (50 mg/l), sodium pyruvate (1 mM) and non-essential amino acids (0.1 mM) under conditions indicated in the figure legends.

### Antibodies and reagents

Sunitinib (#13159) and lonafarnib (#11746) were obtained from Cayman Chemical Company (Ann Arbor, MI). Anti-FNTB (#ab109625) antibody was obtained from Abcam (Cambridge, UK). Anti-β-actin (#3700), SETD2 (23486) and anti-PBRM1 (91894) antibodies were obtained from Cell Signaling Technology, (Danvers, MA). Anti-BAP1(sc-28383) antibody was obtained from Santa Cruz (Dallas, TX).

### Generation of sgRNA library

The custom oligonucleotide array (Supplementary Table 1) was synthesised by Custom Array Inc. Overlapping PCR was performed to incorporate NdeI and XbaI sites to the custom array for subsequent Gibson Assembly (NEB, Ipswich, MA). The PCR products were then cloned into pLX-sgRNA linearised with NdeI and XbaI. pLX-sgRNA was a kind gift from Eric Lander & David Sabatini (Addgene plasmid #50662).^25^ The Gibson library reaction was transformed into XL10-Ultra competent cells. To maintain the complexity of the library, at least 20-fold coverage in library representation was recovered in the transformation and cultured in NYZM + broth for 7 h or until OD600 reached 0.8. Subsequently, deep sequencing (Illumina) was performed to validate the library complexity of the input plasmid and lentivirus pool.

### CRISPR/Cas9-based genome-wide LOF screening

The RNA-guided CRISPR-associated nuclease Cas9 provides an effective means of introducing targeted LOF mutations at specific sites in the genome.^26^ For lentiviral production, we used 293T cells transfected with pCW-Cas9 encoding FLAG-tagged Cas9 nuclease driven by doxycycline-inducible promoter and carrying puromycin resistance marker. 786-O ccRCC cells^9,27^ were infected with Cas9 expressing lentivirus at high multiplicity of infection (MOI) and the stable clones were selected using puromycin. Cas9 induction in the individual clones was assessed by immunoblotting for Cas9 protein. A clone with superior Cas9 induction rate was selected and the cells were subjected to the infection with human CRISPR sgRNA library (containing blasticidin resistance marker) targeting 18,000 genes with 90,000 individual sgRNAs (5 sgRNA per gene) at low MOI < 1. The infected cells were selected using blasticidin and frozen for future manipulations. Deep sequencing on an Illumina Nextseq was used to monitor library composition. Trimmed sequences were aligned to libraries using Bowtie, with zero mismatches tolerated. All alignments from multi-mapped reads were used. Enrichment of individual hairpins was calculated as a median-normalised log-ratio of the fraction of counts. We assigned a four-fold reduction in sgRNAs abundance as significant. A previously established gold standard of 217 genes expected to have growth phenotypes in all cell types (essential) and 947 genes expected to have growth phenotypes in no cell type (nonessential) was used to estimate true positive and false-positive rates. We calculated the number of sgRNAs with significant abundance decrease for all essential and non-essential genes present in our library. False-negative rate was calculated as the ratio of the number of essential genes, for which no decrease in abundance was detected during induction, to the total number of essential genes present in our library. False-positive rate was calculated as the ratio of the number of non-essential genes with a significant decrease in abundance during induction, to the total number of non- essential genes present in our library. We have used the cut-off (no less than 4 sgRNA with no less than 4-fold reduction in abundance) to stratify the candidate list and identify genes contributing to sunitinib resistance.

### Western blot analysis

Cell lysates preparation and western blot analysis were performed as described previously.^28^

### Cell viability and drug interaction analysis

Cell viability was analysed by CellTiter Blue cell viability assay (#G8081) (Promega) as described previously.^10^ Effective doses (EDs) were calculated using XLfit, a Microsoft Excel add-in. The synergistic interaction between sunitinib and lonafarnib was evaluated by the combination index (CI) using CalcuSyn 2.0 software.^29^ CI < 0.1 very strong synergism; 0.1–0.3 strong synergism; 0.3–0.7 synergism; 0.7–0.9 moderate to slight synergism; 0.9–1.1 nearly additive; 1.1–1.45 slight to moderate antagonism; 1.45–3.3 antagonism; >3.3 strong to very strong antagonism.

### Analysis of apoptosis

DNA fragmentation was detected using APO-BRDU kit (#AU1001) (The Phoenix Flow Systems, Inc., San Diego, CA).

### Lysosomal Sunitinib sequestration analysis

786-O cells were plated into 96-well plate (3 × 10^3^ cells per well). Next day, cells were pre-incubated with Lonafarnib (10 μM) for 8 h. Sunitinib (10 μM) were then added to wells with, or without lonafarnib and incubation continued for next 24 h. Image acquisition was performed on an ImageXpress micro automated imaging system (Molecular Devices, Sunnyvale, CA) driven by MetaXpress software. Nine image fields per well were acquired, using three channels to capture matching signal from Hoechst-stained nuclei (DAPI channel, ex 377/50, em 447/60), and vesicles in both fluorescein (for sunitinib) and TRITC (for LysoTracker Red) wavelengths (ex 472/30 em 520/35; ex 525/40, em 585/40 respectively). Epifluorescence images were acquired with a 20x objective (ELWD Plan Fluor, NA 0.45, WD 7.4), using laser auto-focus with a z-offset. Images were analysed using ‘Multiwavelength Scoring’ MetaXpress module for measurement of parameters within each fluorescent channel. Data generated from these analyses were displayed within Acuity Xpress (Molecular Devices).

### siRNA transfection

786-O and PNX0010 cells were transfected with pooled siRNA mix (Cat# SI00031717 and SI00031731) targeting Protein Farnesyltransferase subunit β (FNTB), or non-silencing siRNA (Cat# 0001027281) (Qiagen, Frederick, MD) using Lipofectamine RNAiMAX Transfection Reagent (Thermo Fisher Scientific Inc., Waltham, MA) according to the manufacturer’s instructions. In all, 48 h post-transfection the efficacy of knockdown was validated by Western blotting analysis using specific antibodies.

### Generation of cell lines with deleted CAAX-motifs of Rheb, Rab7a and Rab51

Mutant 786-O cells were generated using pLenti-CRISPRv2 lentiviral vectors expressing sgRNAs targeting C-end of Rheb, Rab7a and Rab25. Targeting sequences are as follows: 5′- ggaggcagaaaaaatggacg (Rheb); 5′- ggacaagaatgaccgggcca (Rab7a) and 5′-gcccaggctggacaggagcc (Rab25). Lentiviral production and infection of 786-O cells were performed as described above. After the selection with puromycin (1 μg/ml), cells were grown at least for 10–14 days and genomic DNA was isolated using Quick-DNA Miniprep kit (Zymo-Research, Irvine, CA) The amplicons spanning the sgRNAs targeting sites were generated by PCR using Ex Taq DNA polymerase, hot-start version (Takara Bio USA, Inc., Mountain View, CA) and specific primers (Supplementary Table S1). The efficacy of knockouts was validated by direct sequencing of amplicons (Supplementary Fig. S2).

### Assessment of in vivo tumour growth

For in vivo studies, 1 × 106 of PNX0010 cells were inoculated s.c. in the flank region of 6-week-old male C.B17/Icr-scid mice (all animal procedures were done in accordance with institutional guidelines on animal care and with appropriate institutional certification; IACUC protocol #13–16). Animals were fed an autoclaved 2018SX diet (Harlan Teklad, Madison, WI) and water ad libitum. Two weeks after the injection of tumour cells, animals were randomly assigned to the control or experimental groups. The sample size *n* = 5 mice/group was selected because the effects of concomitant treatment with sunitinib and linafarnib were evaluated in vivo for the first time in the present study. The mice were treated orally three times per week with: (i) 10% 2-hydroxypropyl-β-cyclodextrin in PBS (vehicle); (ii) sunitinib (40 mg/kg); (iii) lonafarnib (40 mg/kg); (iv) sunitinib (40 mg/kg) and lonafarnib (40 mg/kg) combined. Tumour volumes were calculated using the formula: (volume = 0.52 × (width)^2^ × length) as described previously.^23^ The mice were euthanised using slow introduction of CO_2_ into the chamber. The flow rate for CO_2_ was set to 10–30% displacement of the chamber volume/min.

### Statistical analysis

Statistical analysis was performed using a two-sided Student’s *t*-test. A *p*-value of <0.05 was considered statistically significant.

## Results

### CRISPR/Cas9-based genome-wide LOF screening to identify cellular factors contributing to sunitinib resistance in ccRCC

**Fig. 1:**
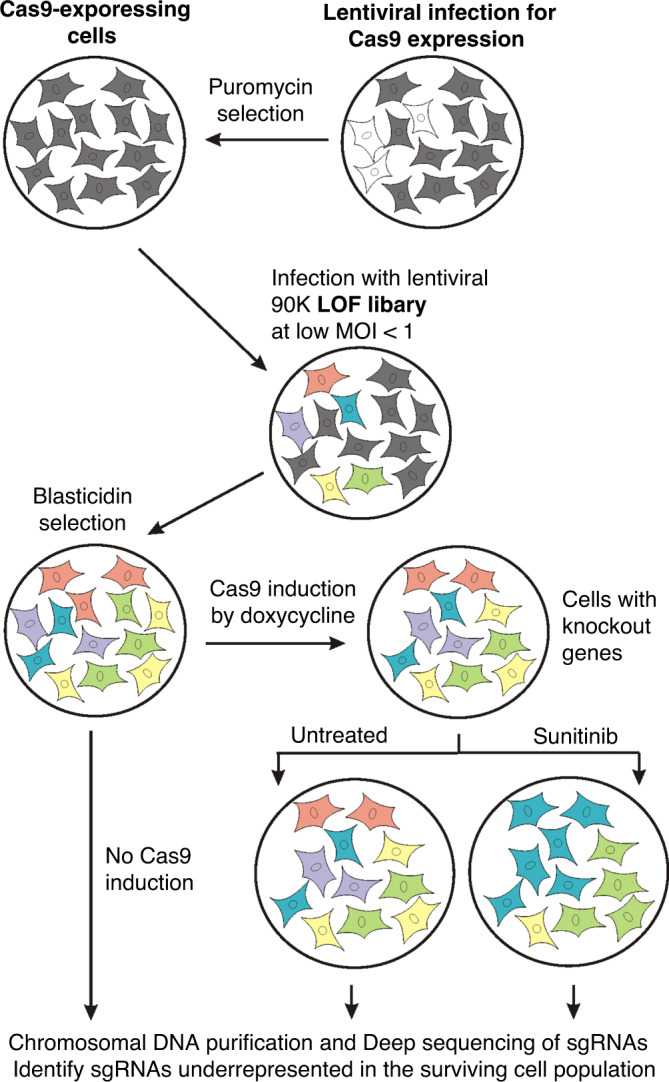
Chart depicting CRISPR/Cas9-based LOF screening strategy to identify molecular targets synthetically lethal with sunitinib treatment.

To identify cellular factors involved in sunitinib resistance, we infected 786-O ccRCC cells with human CRISPR sgRNA library as described in Methods. A brief overview of this strategy is depicted in Fig. 1. Next, we have characterised our library-transformed 786-O cell line and optimised the hit selection parameters using a previously established gold standard set of essential and non-essential genes.^30^ It is expected that after the induction, most of cells harbouring the essential genes knockout should be eliminated, while the non-essential genes should be largely retained. A set of 216 essential genes and 771 nonessential genes present in our library was used to estimate true positive and false-positive rates. By targeting the false-positive rate to be below 1%, we have established the cut-off of no less than 4 sgRNA per gene, with no less than 4-fold reduction in abundance as the threshold for significance (Table 1). Using these stringent criteria, we were able to recover only ~12% of true positives, but this stratification allowed us to optimise the subsequent validation by concentrating on the most reliable candidates first.

**Table 1:** The cutoff criteria for evaluation of true positive and false-positive rates.

		Cutoff (number of sgRNAs with 4-fold reduction)			
	Total	>1	>2	>3	>4
Essential genes	216	138	67	25	5
Non-essential genes	771	105	15	2	0
False-negative rate:		36.1%	69.0%	88.4%	97.7%
False-positive rate:		13.6%	1.9%	0.3%	0.0%

Next, 786-O cells were incubated with sunitinib at 10 μM for 12 days (about six passages). This concentration represents the intratumoural concentration of sunitinib in human tumour specimens (9.5 ± 2.4 μmol/L).^11^ We anticipated that during this time the cells with those knockout genes, which contribute to the resistance to sunitinib would be eliminated from the population. Next, we identified underrepresented sgRNAs and their corresponding gene targets in the surviving cell population. The primers corresponding to sequences flanking the guide in the lentiviral vector included 8-bp bar codes for Illumina-based sequencing. Thus, each sgRNA served as an individual DNA barcode that was used to count the number of cells carrying guides by sequencing. Our search identified a number of genes potentially involved in sunitinib resistance in ccRCC (Supplementary Table S2).^23^

### Identification and validation of druggable molecular factors that are synthetically lethal with sunitinib treatment

**Fig. 2:**
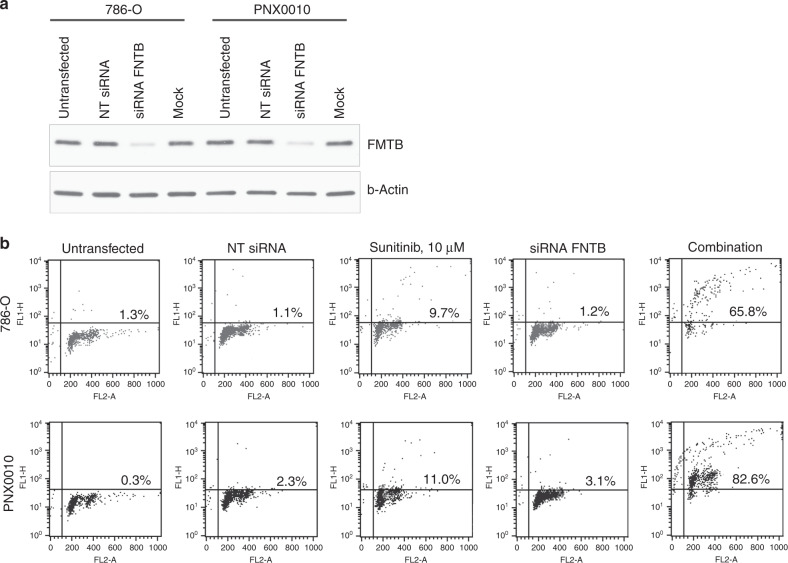
Validation of the role of farnesyltransferase depletion in resistance to sunitinib in ccRCC. **a** Western blot analysis of the indicated ccRCC cells transfected with pooled faranesyltransferase β subunit (FNTB) siRNA. Negative controls include Non targeting siRNA (NT siRNA) and mock transfection (Mock). **b** Analysis of sunitinib-mediated apoptosis in the indicated ccRCC cells transfected with FNTB siRNA and Non targeting siRNA. The percentage of apoptotic cells is indicated in the histograms. The representative data of one out of three independent experiments are presented.

Based on the highest rank of identified hits, we have chosen to focus on the genes, which have not been previously reported to be involved in sunitinib resistance. Our screen identified farnesyltransferase among the top hits. Farnesyltransferase acts as a hetero-dimer comprising from α and β subunits (encoding by genes FNTA and FNTB). To validate whether farnesyltransferase plays a significant role in sunitinib resistance, we performed siRNA mediated depletion of β subunit of this enzyme in 786-O and PNX0010 cells (Fig. 2a). After 24 h, siRNA transfected cells were treated with 10 μM of sunitinib for the next 48 h. As demonstrated in Fig. 2b, the knockdown of farnesyltransferase has dramatically sensitised ccRCC cells to sunitinib-mediated apoptosis.

**Fig. 3:**
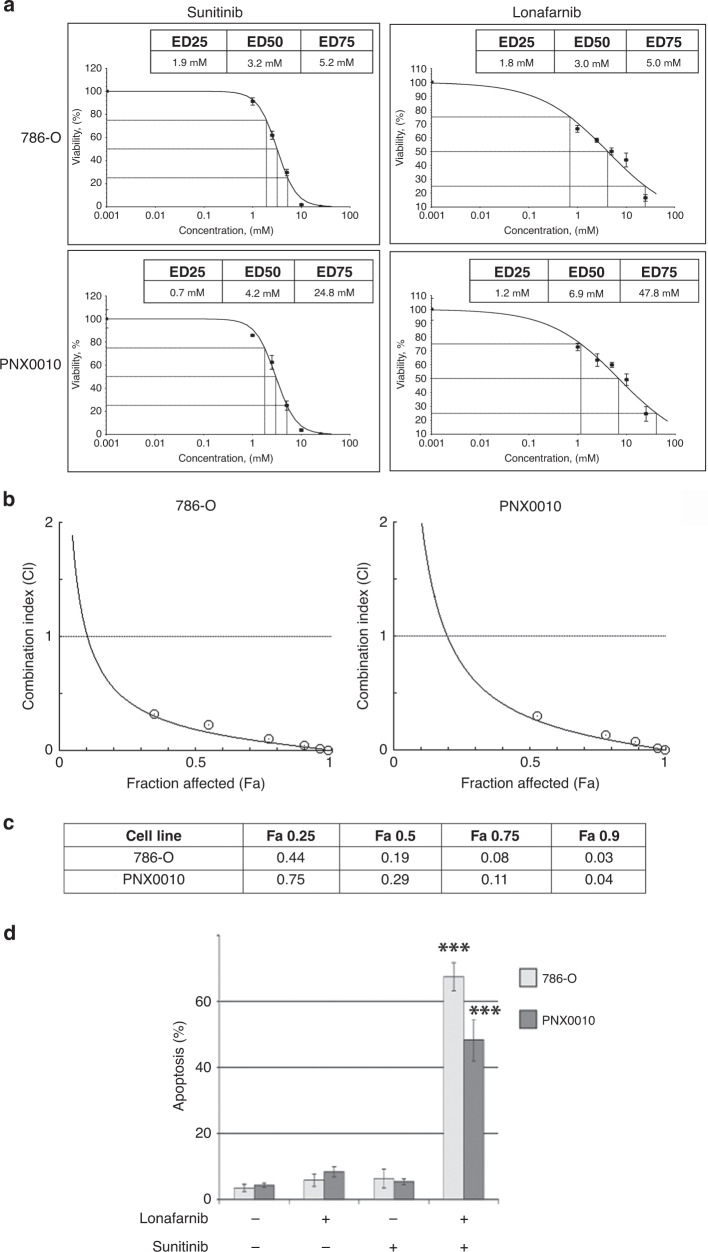
The synergistic effect of combined treatment with sunitinib and lonafarnib on the viability of 786-O and PNX0010 cells. **a** Effective doses (EDs) responses of 786-O and PNX0010 cells to sunitinib and lonafarnib. Cells were treated with either sunitinib or lonafarnib for 72 h. Cell viability was examined by CellTiter Blue assay. EDs (μM) were calculated using XLfit. **b** Combination drug-response curves for the sunitinib plus lonafarnib treatment. 786-O and PNX0010 cells were treated with various combinations of sunitinib and lonafarnib for 72 h. Cell viability was examined by CellTiter Blue assay. **c** The combination index (CI) for the sunitinib plus lonafarnib treatment. The CI was calculated using CalcuSyn 2.0 software as described in Materials and Methods. CI > 1.3: antagonism; CI 1.1–1.3: moderate antagonism; CI 0.9–1.1: additive effect; CI 0.8–0.9: slight synergism; CI 0.6–0.8: moderate synergism; CI 0.4–0.6: synergism; CI 0.2–0.4: strong synergism. **d** 786-O and PNX0010 cells were treated with sunitinib with or without lonafarnib (both at 10 μM) for 24 h. Apoptosis was examined using APO-BRDU kit followed by flow cytometry analysis. Data are presented as the mean ± S.D.

The search through clinicaltrials.gov identified several pharmacological agents inhibiting farnesyltransferase function, including lonafarnib.^31,32^ Viability of 786-O and PNX0010 cell subjected to treatment with lonafarnib and sunitinib was examined using CellTiter Blue viability assay. The effective doses (EDs) of both drugs were assessed using XLfit (Fig. 3a). Next, 786-O and PNX0010 cell were treated with various dosing regimens of lonafarnib and sunitinib to examine a synergistic anti-tumour effect for the combination of these agents. The data analysis using CalcuSyn 2.0 software revealed a high level of synergistic interaction between lonafarnib and sunitinib (Fig. 3b, c). Concomitant treatment with lonafarnib and sunitinib at higher doses resulted in profound DNA fragmentation in 786-O and PNX0010 cells (Fig. 3d).

**Fig. 4:**
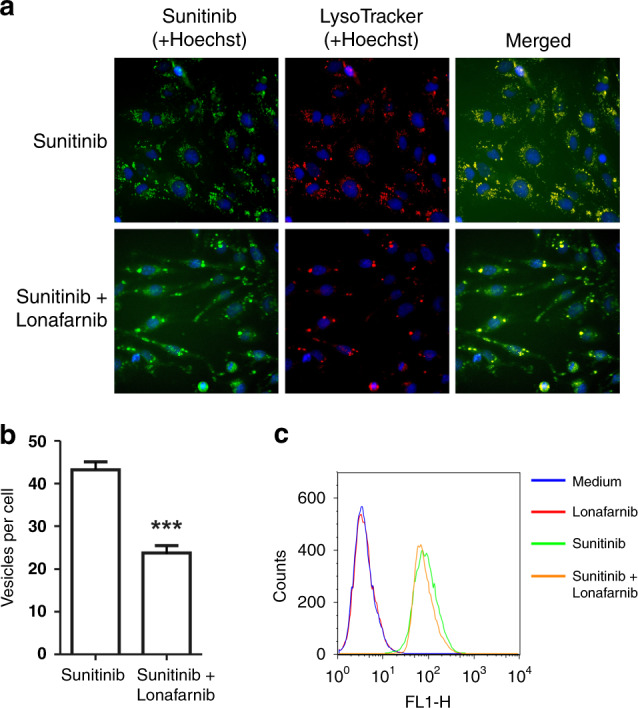
Lysosomal sequestration of sunitinib. **a** 786-O cells were treated with sunitinib with or without lonafarnib, stained with Hoechst and Lysotracker Red, and imaged as described in “Methods”. **b** 786-O cells were treated as described in **a**. The number of sunitinib containing vesicles per cell was calculated as described in Materials and Methods. Data are presented as the mean ± S.D. ***P < 0.0001. **c** The total cellular accumulation of sunitinib was not affected by lonafarnib. 786-O cells were treated as described in **a**. The samples were run on Becton Dickinson FACScan flow cytometer and analysed using FlowJo software.

Lysosomal sequestration of sunitinib limits its intracellular anti-tumour activity.^11^ To further define the mechanism of lonafarnib-mediated sensitisation of tumour cells to sunitinib, we examined whether the lysosomal sequestration of sunitinib can be disrupted by lonafarnib. Sunitinib is a fluorescent compound. Therefore, its intracellular uptake and localisation can easily be monitored. As demonstrated in Fig. 4a, b, treatment with lonafarnib significantly reduced a number of sunitinib-containing lysosomes. Importantly, the total cellular accumulation of sunitinib was not affected by lonafarnib (Fig. 4c).

**Fig. 5:**
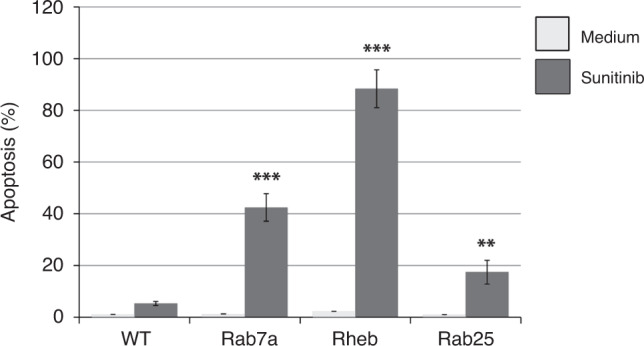
The role of Rab7a, Rab25 and Rheb prenylation in the resistance to sunitinib in ccRCC cells. 786-O and PNX0010 cells were treated with sunitinib at 10 μM for 24 h. Apoptosis was examined using APO-BRDU kit followed by flow cytometry analysis. Data are presented as the mean ± S.D.

Our recent studies demonstrate that co-administration of mTORC1 inhibitors overcomes sunitinib resistance in renal and prostate cancer cells both in vitro and in vivo.^9^ mTORC1 is activated through the direct binding of Rheb,^33^ a GTPase up-regulated in transformed cells.^34^ The farnesylation of Rheb is required for its lysosomal membrane localisation and activation of mTORC1 signalling.^35,36^ FTIs suppress Rheb farnesylation and consequently inhibit mTOR signalling.^37^ To address the individual contributions of post-translational prenylation of Rab7a, Rab25 and Rheb proteins, we generated 786-O cells expressing prenylation-incompetent Rab7a, Rab25 and Rheb proteins using CRISPR/Cas9 mediated truncation of C-terminal fragments of those genes containing CAAX motifs. A panel of generated 786-O cellular sub-lines was then treated with sunitinib. As demonstrated in Fig. 5, cells expressing prenylation-incompetent Rheb protein have shown a robust level of apoptosis after sunitinib treatment. These data suggest the critical role of mTORC1 inhibition in FTI-mediated sensitisation of ccRCC cells to sunitinib. 786-O cells expressing prenylation-incompetent Rab7a protein showed reduced level of apoptotic cell death. Expression prenylation-incompetent Rab25 protein had mild pro-apoptotic effect on 786-O cells treated with sunitinib (Fig. 5).

**Fig. 6:**
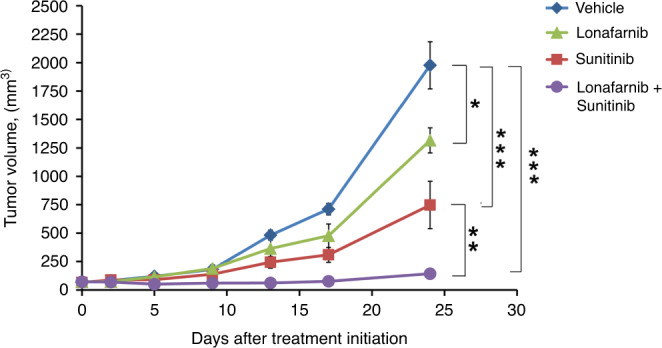
Treatment with lonafarnib circumvents resistance to sunitinib in a xenograft model of human ccRCC. PNX0010 subcutaneous xenograft tumours were established in 6-week-old male C.B17/Icr-scid mice. The mice were treated orally three times per week with: (i) 10% 2-hydroxypropyl-β-cyclodextrin in PBS (control); (ii) sunitinib (40 mg/kg); (iii) lonafarnib (40 mg/kg); (iv) sunitinib and lonafarnib combined. Tumour volumes were calculated as described in “Methods”. Values are means (*n* = 5) ± SD. *P < 0.05; **P < 0.001; ***P < 0.0001.

In light of our encouraging in vitro data, we next examined the anti-tumour effect of sunitinib in combination with lonafarnib using mice bearing PNXC0010 ccRCC xenograft tumours. As demonstrated in Fig. 6, monotherapy with either sunitinib or lonafarnib showed a moderate decrease in the growth of PNXC0010 xenograft tumours. However, combination treatment with sunitinib or lonafarnib resulted in vastly more impressive inhibition of tumour growth among the experimental groups. These results suggest a readily available clinical strategy to circumvent resistance to sunitinib in ccRCC tumours.

## Discussion

CRISPR-based LOF screening represents the state-of-art tools to identify synthetically lethal combinations for targeted cancer therapy. Our present study using CRISPR/Cas9-based genome-wide screen revealed a critical role of FTase as a cellular factor contributing to the resistance of ccRCC cells to sunitinib. The substrate specificity of FTase is determined by the amino acid residues of the CAAX site, in particular the amino acid residue X.^38^ Proteins containing X as methionine or serine exhibit greater affinity for FTase. These are N-Ras proteins containing Cys-Val-Val-Met, K-Ras4a with Cys-Ile-Ile-Met, K-Ras4b with Cys-Val-Ile-Met, and H-Ras with Cys-Val-Leu-Ser.^39^ Indeed, FTIs were first devised to inhibit Ras, however, alternative prenylation of K- and N-Ras hinders these drugs’ ability to affect Ras oncogenes.^40^ Furthermore, clinical effects of FTIs do not appear to be linked to Ras mutations or inhibition of Ras effectors.^41^ Also, our CRISPR/Cas9-based genome-wide screening did not identify Ras and its effectors as central components mediating sunitinib resistance in ccRCC cells. It is therefore likely that other substrates of FTase contribute to the anti-tumour effects of FTase targeted therapeutic agents.

Treatment with TORC1 inhibitors sensitises renal and prostate cancer cells to sunitinib both in vitro and in vivo.^9^ mTORC1 is activated through the direct binding of Rheb,^33^ a GTPase up-regulated in transformed cells.^34^ The farnesylation of Rheb is required for its lysosomal membrane localisation and activation of mTORC1 signalling.^35,36^ FTIs suppress Rheb farnesylation and consequently inhibit mTOR signalling.^37^ Critically, studies by Meier et al. demonstrate that lonafarnib does not inhibit phosphorylation of ERK or AKT but affects phosphorylation of p70S6K, a downstream target of mTOR signalling.^42^ Therefore, FTIs may potentiate the anti-tumour efficacy of sunitinib, at least in part, through two potential mechanisms: (1) suppression of Rheb-dependent mTORC1 activation, and (2) dysregulation of lysosomal sequestration of TKIs. Lysosomal sequestration occurs when a hydrophobic weak base compound enters the lysosome, is protonated in the acid environment, and is unable to cross the membrane. Lysosomal sequestration has been documented for several TKIs including sunitinib, erlotinib, and pazopanib.^11,43^ Interestingly, lysosomal sequestration of sorafenib was observed in hepatocellular carcinoma but not in renal cancer cells.^43,44^ Given that sorafenib does not belong to the same class of hydrophobic weak bases as sunitinib, its lysosomal sequestration could occur via ABC transporter P-glycoprotein (P-gp)-dependent mechanism.^45^ Studies by Colombo et al. demonstrated that treatment with verapamil, a P-gp inhibitor, enhanced the anti-tumour activity of sorafenib and sunitinib, supporting the role of P-gp in TKIs resistance.^44^ Lysosomal sequestration of hydrophobic weak base therapeutics triggers lysosomal biogenesis.^46^ Enhanced lysosomal biogenesis results in augmented lysosomal drug sequestration and multi-drug cross-resistance. Thus, pharmacological inhibition of FTase may reinstate sensitivity to various TKIs through a common mechanism, i.e. dysregulation of lysosomal drug sequestration via inhibition of TKI-mediated lysosomal biogenesis. Indeed, our findings presented in Fig. 4a, b indicate that treatment with lonafarnib impairs lysosomal biogenesis and thus reduces the amount of sunitinib sequestrated in the lysosomal compartment without affecting the total cellular accumulation of sunitinib.

A number of clinical studies investigating the anti-tumour potential of FTIs led to disappointing results, before it was realised that a biochemical redundancy mechanism allows K-Ras activation by geranylgeranylation (catalysed by GGTase I), which takes over the task of Ras prenylation, when FTase is inhibited.^47–49^ Such redundancy may explain the suboptimal clinical efficacy of FTIs (e.g. tipifarnib) for the treatment of pancreatic (90% K-Ras mutations) and lung and colon carcinomas (∼30% K-Ras mutations).^47,50^ Nevertheless, several Phase 2 trials are currently recruiting patients to study the efficacy of tipifarnib against tumours of various origins (NCT03719690, NCT02383927, NCT03496766, NCT02807272 and NCT02535650).

In summary, our current study presents a promising strategy to identify and validate druggable factors involved in the resistance to targeted therapeutics. Our findings suggest a critical role of FTase-dependent cellular factors in the regulation of sunitinib resistance in ccRCC cells. Future studies are required to precisely elucidate mechanisms underlying synergistic interaction between sunitinib and FTIs.

## Supplementary information

Supplementary Materials

## Data Availability

All data are available via the corresponding author.
