# Supplementary material for: CRISPR/Cas9 genome-wide loss-of-function screening identifies druggable cellular factors involved in sunitinib resistance in renal cell carcinoma
Source: Br J Cancer. 2020 Sep 24;123(12):1749–56. doi: 10.1038/s41416-020-01087-x (PMC7723036; doi:10.1038/s41416-020-01087-x)
Supplement: Supplementary file 1 — Supplementary Materials [file 41416_2020_1087_MOESM1_ESM.pdf]

Gene	Primer sequence
Rheb	Fw: 5` AATCGGAGGCTGGTTATG<br><br>Rev: 5` AACTCATTTGGACAGACTCAG
Rab7a	Fw: 5` CTCTCTTCACATTGACCTGG<br><br>Rev: 5` GGACTCTGTGCTCAACTCTC
Rab25	Fw: 5` ATGGGCTCTAAATCTTCTG<br><br>Rev: 5` AAGGAGAAGAGGGAGGAAC

Supplementary table S1. The list of primers used for amplification of genomic regions, spanning sgRNA targeting sites for Rheb, Rab7a and Rab25 genes.

**Gene symbol**
EIF2S3
TTI1
ABCF1
ADCK5
ARL2
B3GNT1
BEGAIN
BRAP
CATSPERD
CEP68
CHD2
DCAF8
DCSTAMP
DDX6
DHX35
DLG5
EEF1A1
EIF6
ERN2
FCER1G

FKBP11
FNTB
GINS1
GINS2
IKBKB
IKBKG
KCNG4
KLHDC7B
LCAT
LRPPRC
LSM11
MDM4
MRGPRD
MYOM3
NFRKB
PTPRS
RPA1
RPAP3
RRAS
RRP1
SERPINB6
SLBP
SLC45A4
SLC5A4
SLC6A12
SPTLC2
SREBF2
TAF10
TAF11
TEKT4
TRIM24
TRIM52
UQCRC1
UTS2R
VPS52
ZSWIM8

Supplementary table S2. The list of genes potentially involved into resistance to sunitinib identified by the screening.

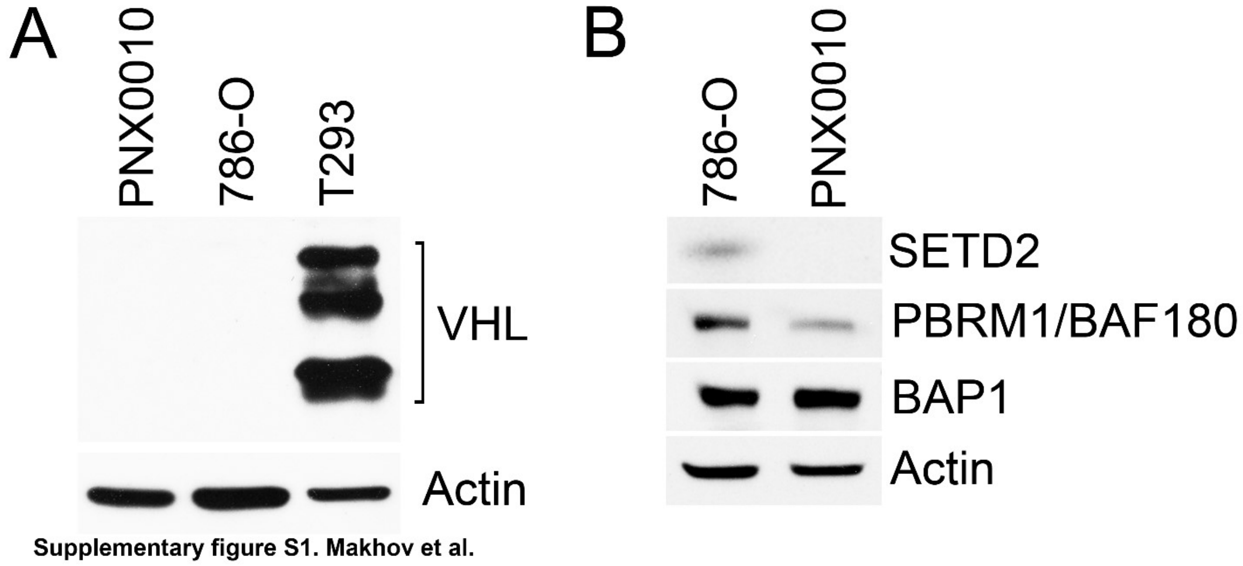

Supplementary figure S1. Western blot analysis of indicated proteins in human embryonic kidney (T293) and ccRCC cell lines (786-O and PNX0010).

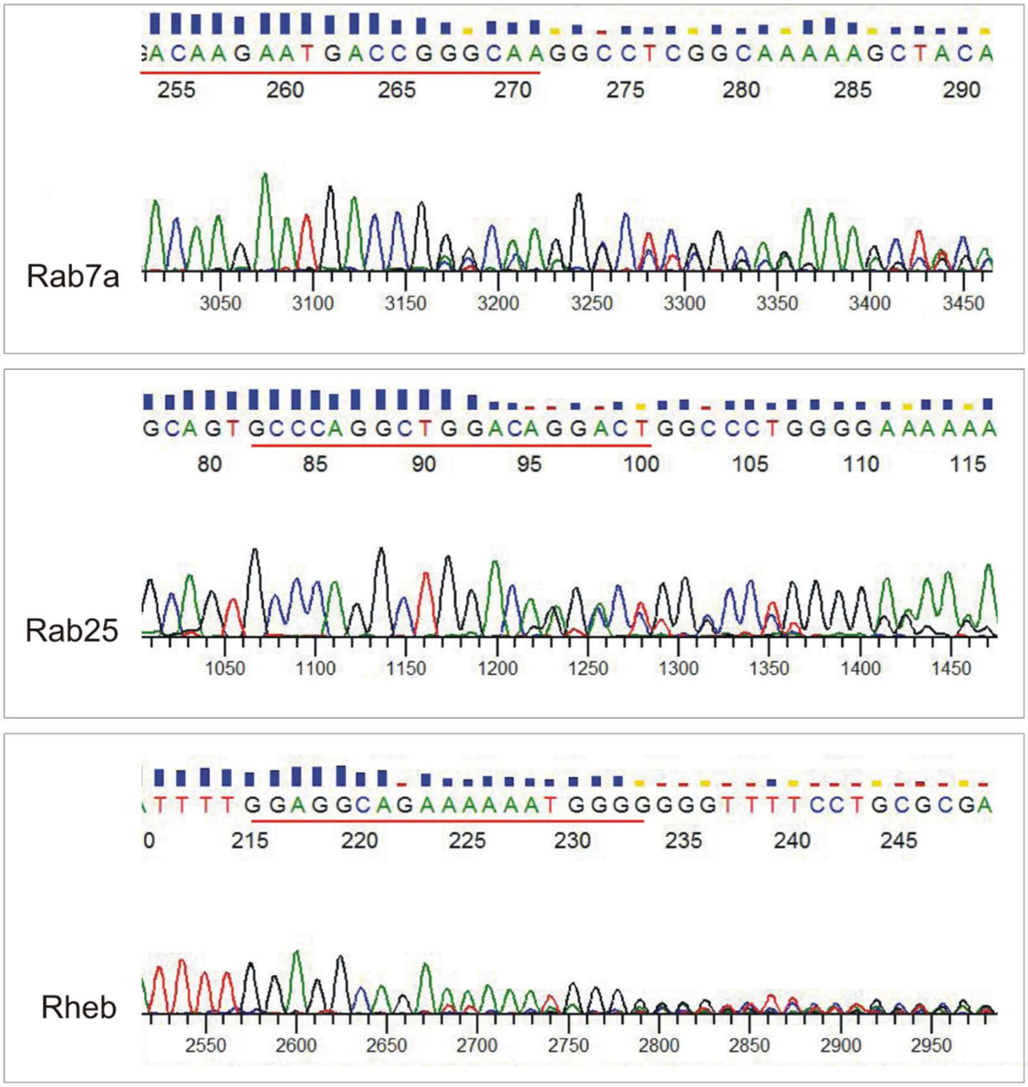

Supplementary figure S2. Makhov et al.

Supplementary figure S2. Validation of the efficacy of CRISPR-Cas9 mediated frameshift mutations upstream of CAAX motifs of indicated genes. Fragments of Sanger sequencing chromatograms of amplicons with sgRNA binding sites (underscored with red) are indicated. Mixed bases (merged individual peaks) occur directly within and downstream of 3` end of sgRNA binding sites.
